# AI and computational methods for reconstructing cell-state-specific gene regulatory networks from single-cell transcriptome and chromatin accessibility data in cancer: methods, applications, and perspectives

**DOI:** 10.3389/fgene.2026.1982712

**Published:** 2026-10-20

**Authors:** Jinying Wang, Fenjuan Chen

**Affiliations:** Department of Internal Medicine, Shaoxing Second Hospital Medical Community General Hospital, Shaoxing, Zhejiang, China

**Keywords:** artificial intelligence, benchmarking, causal validation, chromatin accessibility, gene regulatory networks, single-cell multi-omics, tumor cell states, tumor heterogeneity

## Abstract

Tumor heterogeneity is shaped by genetic, epigenetic, microenvironmental, and stochastic factors, among which cell-state-specific regulatory programs are a major determinant. Gene regulatory networks provide a systems-level model linking transcription factors, cis-regulatory elements, and target genes. Paired single-cell RNA sequencing (scRNA-seq) and single-cell assay for transposase-accessible chromatin using sequencing (scATAC-seq) measure chromatin accessibility and gene expression in the same cell, offering a direct view of regulatory elements and transcriptional output; matched-but-unpaired, imputed, and single-modality designs can extend coverage but introduce additional assumptions and artifacts. This mini-review focuses on AI and computational methods for reconstructing cell-state-specific GRNs from single-cell transcriptome and chromatin accessibility data in cancer. We define inclusion criteria, distinguish same-cell paired, matched-unpaired, imputed, and single-modality designs, and organize methods by inference objective and input requirement. We cover motif/co-accessibility inference, deep integration and graph learning, dynamic and cell-state-specific modeling, and virtual perturbation. We then discuss applications in epithelial-mesenchymal transition, treatment resistance, lineage plasticity, and tumor subtyping, emphasizing an evidence hierarchy from statistical association to directional prediction, *in silico* perturbation, and experimentally supported causal regulation. Finally, we examine benchmarking, limitations, and translational prospects. These approaches may help convert GRNs from descriptive maps into testable models of tumor regulation, but clinical utility still requires prospective validation.

## 1 Introduction

Cancer progression, drug resistance, and relapse are not fully explained by individual driver mutations. Cell-state heterogeneity within tumors and the transitions between states increasingly appear to shape clinical behavior (Groves and Quaranta, 2023; Joanito et al., 2022). Cell states are maintained and switched by GRNs composed of transcription factors, cis-regulatory elements, and target genes (Badia et al., 2023). Reconstructing tumor cell-state-specific GRNs therefore offers a computational basis for predicting resistance, identifying targets, and designing combination strategies.

Classical GRN inference relies on bulk transcriptomes or scRNA-seq alone. Bulk data mix cell types and states, and single-modality expression data cannot distinguish transcription factor expression from transcription factor activity or assign distal enhancers to their target genes (Badia et al., 2023; Loers and Vermeirssen, 2024). Joint profiling technologies, including sci-CAR, SNARE-seq, SHARE-seq, Paired-seq, and 10x Multiome, now capture chromatin accessibility and gene expression from the same cell (Cao et al., 2018; Chen et al., 2019; Ma et al., 2020; Zhu et al., 2019), providing direct evidence for enhancer-promoter-gene relationships.

AI methods are well suited to the high dimensionality, sparsity, and nonlinearity of single-cell multi-omics data. Deep generative models can impute or generate missing modalities (Li et al., 2022; Lan et al., 2023). Graph neural networks explicitly represent relations among genes, peaks, and cells (Ma et al., 2023; Cao and Gao, 2022). Attention mechanisms and foundation models attempt to learn regulatory syntax across cell types (Fu et al., 2025; Hu et al., 2026). Existing reviews mostly organize methods by category or cover broad cancer applications; none centers on cell-state-specific GRN reconstruction from paired scRNA-seq and scATAC-seq data. This review follows that focus, moving from data foundations to AI method families, cancer applications, validation and interpretation, and open problems.

We therefore focus on four levels of evidence that are often conflated in this literature: statistical association, directional regulatory prediction, *in silico* perturbation, and experimentally supported causal regulation. Keeping these levels distinct clarifies what each method can claim and helps avoid overinterpreting network predictions. This review focuses on computational and AI methods for inferring cell-state-specific GRNs from single-cell transcriptome and chromatin-accessibility data in cancer. We included studies that resolved single cells or nuclei, used transcriptomic and/or chromatin-accessibility measurements, and explicitly inferred regulatory networks or perturbation effects relevant to cancer cell states. Same-cell paired multi-omics is our primary focus; matched-unpaired, imputed/transferred, and single-modality methods are included when they provide useful context or address a core inference problem. We excluded methods based only on bulk data or on sequence-based predictions without a single-cell regulatory component. Preprints are identified as such.

Several recent reviews cover single-cell GRN inference, multimodal integration, and foundation models. These are valuable but differ from the present review in scope, cancer focus, and treatment of paired, unpaired, and imputed data. Our aim is to complement them by providing a practical input-design taxonomy, an explicit evidence hierarchy, guidance for benchmarking and method selection, and a cancer-focused synthesis of applications. These themes organize the remainder of the review.

## 2 Paired single-cell multi-omics data: technical foundations and challenges

sci-CAR, reported in 2018, measured chromatin accessibility and mRNA in thousands of single cells and linked cis-regulatory sites to target genes through covariance (Cao et al., 2018). In the original study, sci-CAR was applied to a dexamethasone treatment time series and to thousands of cells from adult mouse kidney, and it reconstructed cell-type accessibility profiles from RNA-defined cell types. SNARE-seq later combined droplet-based single-nucleus profiling with higher throughput (Chen et al., 2019). It generated joint profiles from neonatal and adult mouse cerebral cortex and connected promoter accessibility dynamics with transcription during neurogenesis. SHARE-seq scaled the approach across tissues and introduced the concept of chromatin potential, in which accessibility changes predict gene expression changes that have not yet occurred (Ma et al., 2020). Using tens of thousands of joint profiles from mouse skin, SHARE-seq identified cis-regulatory interactions and defined a computational strategy for linking regulatory elements to genes. Paired-seq extended throughput to millions of cells and supported cell-type-specific regulatory analysis in complex tissues (Zhu et al., 2019). In adult mouse cerebral cortex and fetal forebrain, Paired-seq inferred putative target genes of candidate enhancers and reconstructed lineage trajectories. These methods established the same-cell, two-modality data paradigm that underlies most current GRN inference from paired multi-omics. The result is a data type that neither modality provides alone.

Deep generative models offer a computational alternative when paired profiling is costly or one modality is missing. scMVP uses a multi-view variational autoencoder to represent SNARE-seq, sci-CAR, Paired-seq, SHARE-seq, and 10x Multiome data for clustering, trajectory inference, and cis-regulatory element identification (Li et al., 2022). scMOG generates *in silico* scATAC profiles from scRNA-seq or the reverse direction, and in lymphoma data the generated ATAC profiles identify tumor samples at least as well as measured ones (Lan et al., 2023). Such methods let researchers reuse historical single-modality datasets and reduce the burden of paired data collection.

The resulting data also pose analytical challenges. Paired single-cell data bring new information and new difficulties. Dropout and sequencing depth vary widely, and scATAC-seq is especially sparse, which lowers the signal-to-noise ratio of peak-to-gene linkage (Badia et al., 2023; Loers and Vermeirssen, 2024). Dropout is not random: genes with low expression are detected less reliably, and transcription factors that are expressed at low levels may be absent from the measured matrix even when they regulate a target gene. Batch and platform differences can obscure genuine cell-state signals and require careful integration and normalization (Cao and Gao, 2022). When cells from different samples or centers are combined, technical variation can create artificial clusters or hide real ones. Distal enhancer-promoter relationships, motif degeneracy, and allele-specific effects make it difficult to infer direction from co-accessibility or expression correlation alone (Loers and Vermeirssen, 2024; Zhang et al., 2022). An accessible peak near a gene may be unrelated to that gene if the enhancer contacts a different promoter in the three-dimensional genome. Tumor cells also occupy continuous trajectories, and cell cycle, differentiation state, and microenvironment signals are mixed in the same sample; ignoring this context yields population-averaged networks that do not correspond to any real cell state (Groves and Quaranta, 2023; Perez-Gonzalez et al., 2026).

Conceptually, paired data change the inference problem. Accessibility reflects regulatory potential, expression reflects output, and the relationship between the two is neither instantaneous nor deterministic. A promoter can become accessible before its gene is induced, and an enhancer can be occupied without changing steady-state expression. GRN methods should therefore model time lags, indirect effects, and context-dependent wiring instead of treating single-time-point correlation as regulation.

It is also important to distinguish four types of input data. Same-cell paired data measure transcriptome and chromatin accessibility in the same cell, as in 10x Multiome, SHARE-seq, SNARE-seq, sci-CAR, and Paired-seq. Matched-but-unpaired data measure the two modalities in separate cells from the same sample or donor and require anchoring or label transfer. Imputed or transferred designs generate one modality from the other, as in scMVP and scMOG, and can introduce model-dependent artifacts. Single-modality designs use scRNA-seq or scATAC-seq alone, often with external priors. Table 1 reports these categories explicitly.

**TABLE 1:** Representative methods for GRN inference from paired single-cell multi-omics data.

Method	Data type	Modeling strategy	Cell-state resolution	Validation	Input design	Directionality/perturbation	Main limitations
SCENIC+ (Bravo Gonzalez-Blas et al., 2023)	scRNA + scATAC	Co-expression, TF motifs, enhancer-gene linkage	Cell groups/states	Multi-species, multi-dataset benchmarks	Same-cell paired or paired input	Directed prediction; perturbation support varies	See method-specific limitations in the text
scREG (Duren et al., 2022)	scRNA + scATAC	Cis-regulatory potential dimension reduction	Subgroups	Network inference accuracy, GWAS enrichment	Same-cell paired or paired input	Directed prediction; perturbation support varies	See method-specific limitations in the text
FigR (Kartha et al., 2022)	scATAC + scRNA	DORCs, peak-gene pairing	Cell types/stimulus states	Immune stimulation experiments	Same-cell paired or paired input	Directed prediction; perturbation support varies	See method-specific limitations in the text
DIRECT-NET (Zhang et al., 2022)	scRNA + scATAC, or scATAC only	Gradient boosting	Subgroups/dynamic	Independent functional genomics data	Paired or scATAC-only	Directed prediction; perturbation support varies	See method-specific limitations in the text
LINGER (Yuan and Duren, 2025)	scRNA + scATAC + external atlases	Neural network with motif manifold regularization	Cell types/contexts	Accuracy gains across datasets, case-control TF activity	Same-cell paired or paired input	Directed prediction; perturbation support varies	See method-specific limitations in the text
scMultiomeGRN (Xu X et al., 2025)	scRNA + scATAC	Cross-modal attention and neighbor aggregation	Cell-specific	Multi-omics benchmarks	Same-cell paired or paired input	Directed prediction; perturbation support varies	See method-specific limitations in the text
DeepMAPS (Ma et al., 2023)	scMulti-omics	Heterogeneous graph transformer	Cell types	Clustering and network benchmarks	Same-cell paired or paired input	Directed prediction; perturbation support varies	See method-specific limitations in the text
GLUE (Cao and Gao, 2022)	scRNA + scATAC and other omics	Graph-linked unified embedding	Cell types/atlases	Large-scale integration benchmarks	Same-cell paired or paired input	Directed prediction; perturbation support varies	See method-specific limitations in the text
Dictys (Wang et al., 2023b)	scRNA + scATAC	Footprinting, stochastic processes, probabilistic model	Dynamic/contexts	Developmental continuum validation	Same-cell paired or paired input	Directed prediction; perturbation support varies	See method-specific limitations in the text
inferCSN (Li et al., 2025)	scRNA	Pseudotime-based inference	Cell types and states	Immune regulation datasets	Single-modality (scRNA)	Directed prediction; perturbation support varies	See method-specific limitations in the text
GeneLink (Zhang et al., 2025)	scRNA	Cell-type-specific modeling	Cell types	Multiple datasets	Single-modality (scRNA)	Directed prediction; perturbation support varies	See method-specific limitations in the text
ScReNI (Xu. et al., 2025)	scRNA + scATAC, paired or unpaired	Nearest-neighbor and modified random forest	Single cells	Network clustering and regulator identification	Context-dependent	Directed prediction; perturbation support varies	See method-specific limitations in the text
CellOracle (Kamimoto et al., 2023)	scRNA + scATAC	GRN + *in silico* TF perturbation	Cell states	Developmental and hematopoietic phenotype recapitulation	Same-cell paired or paired input	Directed prediction; perturbation support varies	See method-specific limitations in the text
Pando (Fleck et al., 2023)	scRNA + scATAC + binding site prediction	Multi-omics GRN framework	Cell states	Pooled perturbation in organoids	Same-cell paired or paired input	Directed prediction; perturbation support varies	See method-specific limitations in the text
GenKI/scTenifoldKnk (Osorio et al., 2022; Yang et al., 2023)	scRNA	Graph autoencoder/manifold-alignment virtual knockout	Cell groups	Recapitulation of real knockout phenotypes	Single-modality (scRNA)	Directed prediction; perturbation support varies	See method-specific limitations in the text
scPRAM (Jiang et al., 2024)	scRNA perturbation data	Attention and optimal transport	Cell types	Multi-perturbation, cross-species prediction	Perturbation scRNA	Directed prediction; perturbation support varies	See method-specific limitations in the text
scMTNI (Zhang et al., 2023)	scRNA + scATAC	Multi-task learning with lineage structure	Lineage/cell types	Simulated and real lineage data	Same-cell paired	Directed; no perturbation	Requires lineage structure; cell-type labels
scMEGA (Li et al., 2023)	scRNA + scATAC	Enhancer-to-promoter association pipeline	Dynamic/cell states	Case study in myocardial infarction	Same-cell paired	Directed; no perturbation	Enhancer assignment; dynamic assumptions
STREAM (Li et al., 2024)	scRNA + scATAC	Steiner forest + biclustering + submodular optimization	Cell states/lineages	TF/enhancer-gene benchmarks	Same-cell paired	Directed; no perturbation	Optimization complexity; paired data

## 3 AI frameworks for GRN inference

According to modeling strategy, current methods fall into four families: motif- and co-accessibility-based inference, deep integration and graph learning, dynamic and cell-state-specific inference, and virtual perturbation. The flow from paired multi-omics to cell-state-specific GRNs, experimental validation, and cancer applications is shown in Figure 1.

**FIGURE 1:**
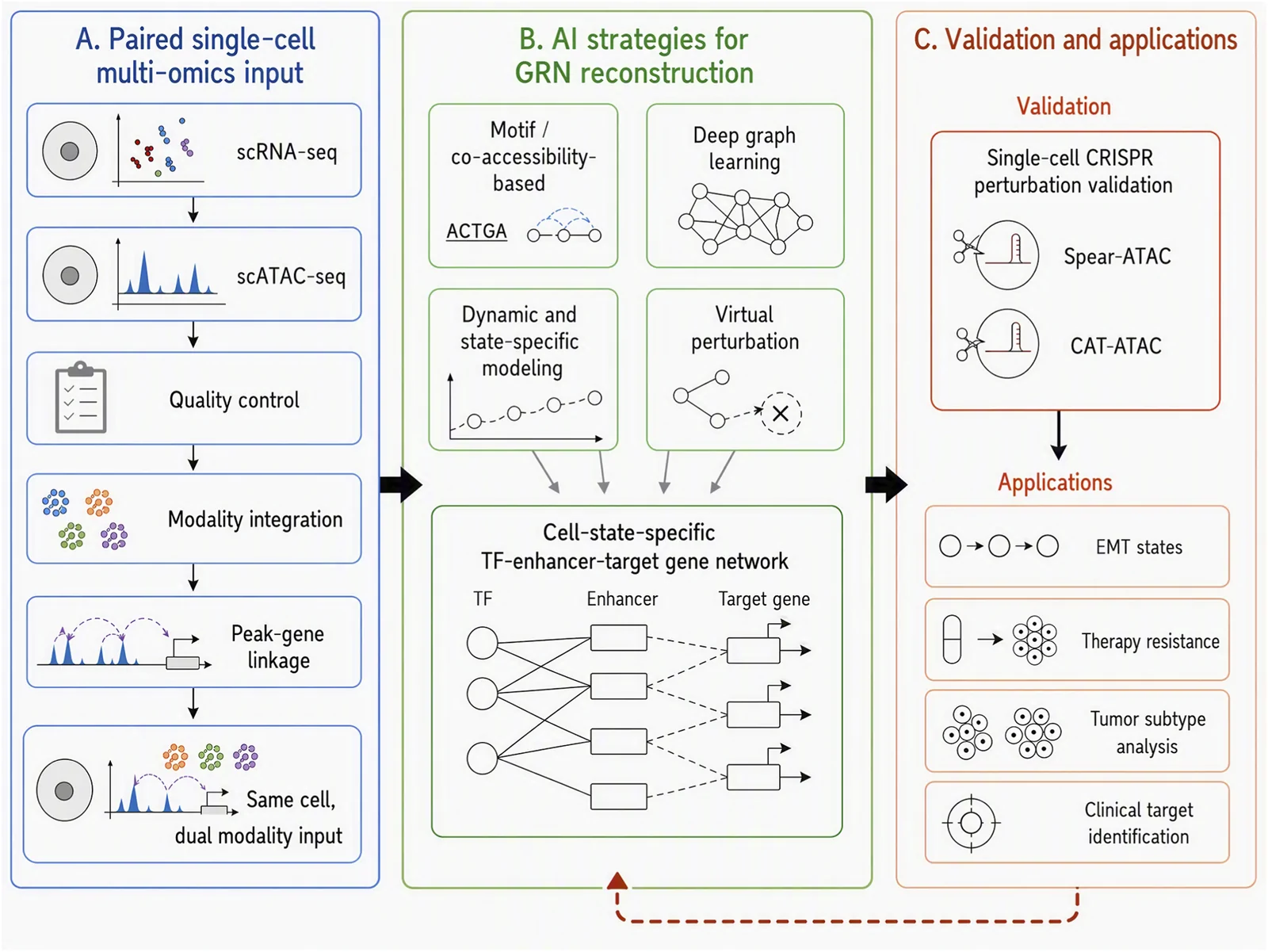
Overview of the pipeline for reconstructing cell-state-specific gene regulatory networks. **(A)** Paired single-cell multi-omics input (scRNA-seq and scATAC-seq), quality control, modality integration, and peak-gene linkage. **(B)** AI strategies for GRN reconstruction, including motif/co-accessibility-based inference, deep graph learning, dynamic modeling, virtual perturbation, and construction of cell-state-specific networks. **(C)** Validation by single-cell CRISPR methods (e.g., Spear-ATAC, CAT-ATAC) and downstream applications in EMT states, therapy resistance, tumor subtype analysis, and clinical target identification.

Each family makes different assumptions about what counts as evidence. Motif-based methods assume that sequence grammar is interpretable and conserved; graph methods assume that local co-variation and global topology carry regulatory signal; dynamic methods assume that trajectory information resolves direction; perturbation methods assume that edge deletion approximates gene loss. These assumptions determine the typical failure modes, and method choice should depend on the data structure and the biological question.

The first family relies on motifs and co-accessibility. SCENIC reconstructs GRNs from scRNA-seq by combining co-expression modules, transcription factor motif enrichment, and regulon activity scoring, and uses the resulting regulons to identify cell states (Aibar et al., 2017). The original SCENIC workflow was applied to tumor and brain datasets and showed that cis-regulatory analysis can guide the identification of transcription factors and cell states. SCENIC + extends this framework to paired multi-omics data and infers enhancer-driven GRNs, using a curated and clustered motif collection of more than 30,000 motifs to improve transcription factor identification (Bravo Gonzalez-Blas et al., 2023). It predicts enhancers, candidate upstream transcription factors, and target genes in a single framework, and it was benchmarked on melanoma cell states, human peripheral blood mononuclear cells, ENCODE cell lines, and *Drosophila* retinal development. The same predictions can be used to compare conserved regulatory programs across species and to examine how regulatory programs change along differentiation trajectories.

Other methods start from peak-to-gene relationships. scREG defines cis-regulatory potential and uses it for dimension reduction and the construction of subpopulation-specific cis-regulatory networks (Duren et al., 2022). This concept combines gene expression and chromatin accessibility in a way that reflects the regulatory link between an element and a gene. FigR pairs scATAC-seq with scRNA-seq, identifies domains of regulatory chromatin (DORCs) in immune stimulation responses, and infers GRNs (Kartha et al., 2022). FigR was applied to resting and stimulated human blood cells and showed that chromatin accessibility and gene expression change on short timescales during immune activation. DIRECT-NET uses gradient boosting to identify genome-wide cis-regulatory elements and their target genes from paired data or scATAC-seq alone, and can reveal subpopulation-specific and dynamic regulatory connections (Zhang et al., 2022). It was validated with independent functional genomics data and improved the accuracy of cis-regulatory element-to-gene inference compared with existing methods. MICA applies mutual information together with chromatin accessibility refinement in settings with very few cells, such as early human embryos, and improves the reproducibility of network inference (Alanis-Lobato et al., 2024). MICA captures non-monotonic dependencies and feedback loops that linear correlation methods miss. These methods use interpretable biological inputs but have limited capacity for nonlinear relations and global regulatory structure.

A second family uses deep integration and graph learning. Deep learning provides stronger representation learning for high-dimensional sparse multi-omics data. LINGER incorporates atlas-scale external bulk data and transcription factor motif priors through manifold regularization, infers GRNs from paired single-cell data, and transfers the network to expression-only samples to estimate transcription factor activity (Yuan and Duren, 2025). LINGER achieved higher accuracy than existing methods in the reported benchmarks and enabled interpretation of disease-associated variants and genes in genome-wide association studies. scMultiomeGRN builds modality-specific neighbor aggregators and cross-modal attention modules to learn transcription factor representations and construct cell-specific regulatory networks (Xu J et al., 2025). The cross-modal attention mechanism allows the model to combine transcriptomic and epigenomic evidence when deciding whether a transcription factor regulates a target gene. DeepMAPS models single-cell multi-omics as a heterogeneous graph and uses a multi-head graph transformer to learn cell-gene relations in local and global contexts, producing cell-type-specific networks in lung cancer and other datasets (Ma et al., 2023). GLUE explicitly models cross-omics regulatory relations through graph-linked unified embedding, supporting integration and regulatory inference at the scale of millions of cells (Cao and Gao, 2022). GLUE was benchmarked on triple-omics integration and multi-omics human cell atlas construction, and it can correct previous cell annotations in large atlases.

Graph neural networks are a natural fit for gene-peak-cell tripartite relations. Heterogeneous attention models embed scRNA-seq and scATAC-seq nodes in a unified space and infer regulatory edges (Cai et al., 2024). MultiCausGRN introduces directed prior regulatory edges into graph attention learning, improving stability and interpretability when samples are limited (Alkhateeb and Awad, 2026). ScReNI infers single-cell-specific networks with nearest-neighbor search and a modified random forest, and identifies cell-enriched regulators (Xu. et al., 2025). DeepRIG uses a graph autoencoder to learn global regulatory structure and generates cell-type-specific networks in triple-negative breast cancer (Wang J. et al., 2023). scSAGRN incorporates spatial association into multi-omics network inference and improves peak-gene and transcription factor-gene recovery in benchmark evaluations (Ren et al., 2025). Graph methods differ in how the graph is constructed, how neighbor information is aggregated, and whether priors are used to orient edges. They exploit both local co-variation and global topology, but their performance depends heavily on the quality of priors and benchmark labels.

A third family addresses dynamics and cell-state specificity. Tumor cell states are continuous and plastic, and static networks miss regulatory rewiring. Dictys combines multi-omics footprinting, stochastic process networks, and probabilistic models to infer dynamic GRNs along developmental continua (Wang L. et al., 2023). Dictys incorporates context-specific transcription factor footprinting and models single-cell RNA read counts probabilistically, which improves reconstruction accuracy and reproducibility in the reported benchmarks. inferCSN reorders cells with pseudotime and builds cell type- and state-specific networks (Li et al., 2025). Because cells are unevenly distributed along pseudotime, inferCSN takes this distribution into account when estimating regulatory relationships. GeneLink infers cell-type-specific GRNs from single-cell transcriptomes (Zhang et al., 2025). scTIGER detects condition-specific network rewiring from paired case-control scRNA-seq datasets using attention-based convolutional networks (Dautle et al., 2023). scTIGER was applied to prostate cancer and neuronal datasets, where it identified condition-specific regulatory networks for known drivers. Inferelator 3.0 scales to millions of cells and learns cell-type-specific networks that are aggregated into a shared network (Skok Gibbs et al., 2022). Together these methods shift GRN inference from one network per dataset toward one network per cell state.

State-specific inference also raises the question of what a network means when it changes continuously. The single-GRN-per-state view is an approximation; the useful unit may be a regulatory module stable across neighboring states. Dynamic methods should therefore report not only edges but also how edge weights and programs change, and evaluation should ask whether inferred dynamics predict intervention outcomes.

The fourth family generates candidate causal hypotheses through virtual perturbation. Correlation-based networks cannot separate regulation from co-occurrence. Virtual perturbation methods remove or alter edges in a network to simulate knockout, generating causal candidates without perturbation experiments. CellOracle infers GRNs from paired multi-omics data and performs *in silico* transcription factor perturbation, recapitulating known phenotypes in mouse and human hematopoiesis and zebrafish embryogenesis (Kamimoto et al., 2023). In zebrafish, CellOracle predicted a previously unreported notochord phenotype and identified an axial mesoderm regulator that was later validated. Pando integrates multi-omics and transcription factor binding predictions in human brain organoids and validates regulators such as GLI3 with pooled genetic perturbation (Fleck et al., 2023). Pando distinguishes transcription factors that control the abundance of cell fates from those that affect cell state after differentiation. GenKI and scTenifoldKnk use a variational graph autoencoder and manifold alignment, respectively, to perform virtual knockout from wild-type data alone (Yang et al., 2023; Osorio et al., 2022). GenKI removes all edges of the target gene from the network and compares latent representations of wild-type and virtual knockout data, while scTenifoldKnk aligns the reduced network to the original network and identifies differentially regulated genes. scPRAM predicts perturbation responses for unseen cell types with an attention mechanism, supporting extrapolation across individuals and species (Jiang et al., 2024). Table 1 compares the representative methods discussed here by data type, modeling strategy, cell-state resolution, and validation approach. Methods also differ in what they consider regulatory evidence, such as motif conservation, graph topology, trajectory dynamics, or perturbation response. These differences should guide method selection.

scMEGA performs end-to-end enhancer-based GRN inference from single-cell multi-omics data, integrating modalities, trajectory analysis, enhancer-to-promoter association, network analysis, and visualization; its case study examined gene regulatory networks controlling myofibroblast activation in human myocardial infarction (Li et al., 2023). STREAM uses a Steiner forest framework with biclustering and submodular optimization to infer enhancer-driven GRNs from jointly profiled single-cell transcriptome and chromatin-accessibility data, and reports improved TF recovery, TF-enhancer linkage, and enhancer-gene relation discovery in Alzheimer’s disease and lymphoma datasets (Li et al., 2024). scMTNI uses multi-task learning to infer cell-type-specific GRNs on linear and branching cell lineages from scRNA-seq and scATAC-seq data (Zhang et al., 2023).

Evaluating GRN methods requires separating edge-recovery performance from downstream biological utility. Edge-recovery metrics such as AUROC, AUPRC, early precision, odds ratio, and statistical confidence are sensitive to class imbalance, label completeness, reference-network choice, and thresholding. Downstream evaluation should assess TF-target recovery, enhancer-gene recovery, perturbation-response prediction, recovery of known regulatory programs, cross-dataset or cross-cell-state generalization, stability across seeds and subsampling, calibration, and negative controls. ENCODE (ENCODE Project Consortium, 2012) and ChIP-seq provide context-specific TF-binding evidence but are incomplete and cell-type biased. DREAM (Marbach et al., 2012) and BEELINE (Pratapa et al., 2020) offer community benchmarks but were largely designed for earlier settings. Perturb-seq and single-cell CRISPR screens (Dixit et al., 2016; Replogle et al., 2022) provide functional readouts, although they cover limited genes, states, and contexts. Benchmarks should therefore state what they can and cannot establish, report label coverage and an observable metric ceiling, and test incremental value over simple co-expression or gene-level baselines. Donor- or cell-type-aware splits, code availability, and sensitivity to preprocessing and hyperparameters should also be reported.

Throughout this review, we use four terms with distinct meanings. Statistical association describes a non-directional dependency between genes, peaks, or regulators. Directional regulatory prediction adds an inferred direction but remains model-dependent. In silico perturbation generates a hypothesis by altering a network or latent state and does not by itself establish causality. Experimentally supported causal regulation requires orthogonal perturbation, such as CRISPR knockout, CRISPR interference, or Perturb-seq, ideally with independent validation. Virtual perturbation and attention-derived edge scores should therefore be treated as hypothesis-generating evidence, not as causal proof.

Method selection should be guided by both the available data and the question being asked. When same-cell paired data are available and the goal is a static cell-state network, motif and co-accessibility methods such as SCENIC+, scREG, scMEGA, or STREAM are appropriate. For lineage or trajectory dynamics, scMTNI, Dictys, and inferCSN are better aligned with the question. When only scRNA-seq is available, GeneLink, inferCSN, scTIGER, and virtual-knockout methods can be used, but directionality and enhancer linkage require additional priors. When only scATAC-seq is available, DIRECT-NET or motif-based approaches can generate hypotheses, but transcriptome-level consequences remain indirect. Matched-unpaired or imputed designs require explicit anchoring and sensitivity analyses. When perturbation prediction is the goal, CellOracle, Pando, GenKI, scTenifoldKnk, and scPRAM should be paired with orthogonal validation. Foundation models can improve transfer and representation learning, but their regulatory outputs should be compared with simple baselines and validated experimentally. The decision logic is summarized in Figure 2.

**FIGURE 2:**
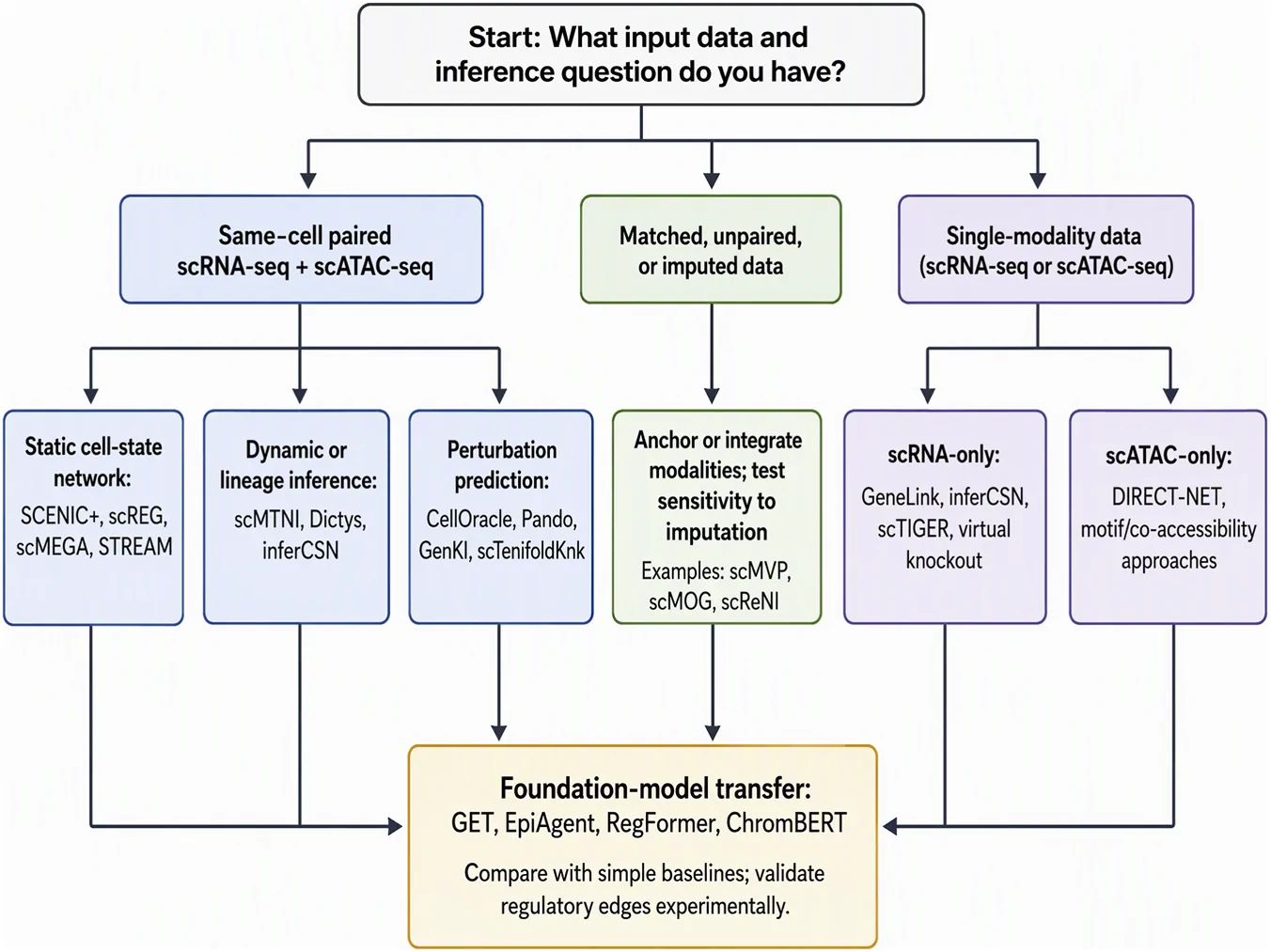
Decision framework for selecting a GRN inference strategy.

## 4 Applications of cell-state-specific GRNs in cancer

These applications show how network inference can help explain tumor behavior. Epithelial-mesenchymal transition (EMT) is a canonical example of tumor cell-state plasticity. In a mouse model of skin squamous cell carcinoma, paired scRNA-seq and scATAC-seq delineated the transcriptomic and chromatin profiles of distinct EMT states, and CRISPR/Cas9 loss-of-function experiments showed that Klf5 and Pitx1 control early EMT and are required for metastasis, whereas Nfatc1 and Creb3l1 act at later stages (Perez-Gonzalez et al., 2026). In melanoma, regulatory-network-based quantitative state diagrams revealed multiple resistance routes, including dedifferentiation and a hyperpigmented phenotype, and *in silico* perturbations identified combination strategies that reduce occupancy of drug-resistant states (Pillai et al., 2022).

These examples show that paired data can identify both the timing of regulatory control and the specific transcription factors that drive state transitions. The same regulatory logic can be tested in other carcinomas, where EMT states have been associated with invasion, metastasis, and resistance to targeted therapy.

Resistance and plasticity are among the most clinically important applications. GRN topology itself can encode treatment escape. In patient-derived glioma stem-like cells, single-cell regulatory network inference showed that network topology determines whether resistant or sensitive cells move through distinct intermediate states under drug treatment, and simulations guided siRNA or combination treatments that increase killing (Park et al., 2024). In melanoma, a TCF4-dependent GRN confers resistance to immunotherapy (Pozniak et al., 2024). On the experimental side, Spear-ATAC couples pooled CRISPR knockdowns with single-cell chromatin accessibility and characterized the temporal dynamics of epigenetic responses across 414 knockdown populations in cancer cells (Pierce et al., 2021). CAT-ATAC captures transcriptome, chromatin accessibility, and single guide RNA (sgRNA) identity from the same cell, allowing GRN construction under drug and genetic perturbations (Shevade et al., 2025).

The combination of network prediction and perturbation readout is especially valuable for resistance, because it can distinguish regulators that are merely correlated with resistance from those that are functionally required. This distinction is difficult to make with expression data alone and is one of the main arguments for treating GRN inference and perturbation screening as a single workflow.

Paired multi-omics has also refined tumor subtype and lineage definitions and uncovered cell-state-specific regulatory programs across solid and hematologic tumors. In colorectal cancer, single-cell and bulk integration identified two epithelial tumor cell states and refined the consensus molecular classification (Joanito et al., 2022); an unsupervised neural network approach further integrated colorectal cancer scRNA-seq and scATAC-seq to construct GRNs within copy-number-defined gene blocks (Cui et al., 2023). In hepatocellular carcinoma, cell fate inference combined with SCENIC identified immune-activated, metabolic, and proliferative terminal states, and the proliferative state signature was associated with worse overall survival (Deng et al., 2026). In pediatric ETV6-RUNX1-positive acute lymphoblastic leukemia, transcription factor activity along the B-lineage trajectory identified targetable tumor cell states during chemotherapy (Mehtonen et al., 2020). A large single-cell atlas of acute myeloid leukemia revealed age-related GRN signatures in t (8; 21) AML and validated an enhancer-driven network with paired multi-omics data (Whittle et al., 2026). Single-nucleus multi-omics in lung adenocarcinoma delineated epigenetic programs linked to tumor progression (Kim et al., 2026). Chromatin analysis in clear cell renal cell carcinoma identified a tumor-intrinsic interferon program associated with BAP1 loss and poor prognosis (Camp et al., 2026). In prostate cancer, FOXA1 mutations rewire nascent transcription factor networks and drive lineage transformation (Ladewig et al., 2026). In gastric cancer, single-cell multi-omics showed plasticity of isthmus stem cells during carcinogenesis (Liu et al., 2025).

Across these studies, cell-state-specific GRNs provide interpretable molecular classifiers and point to state-specific candidate targets. They also illustrate that the same computational strategy, when applied to different tumor types, often identifies distinct core regulators that reflect tissue of origin and oncogenic context.

The same logic extends to the tumor microenvironment. Cell-state-specific GRNs are not confined to tumor cells. Single-cell multi-omics in triple-negative breast cancer resolved a myofibro-inflammatory program in cancer-associated fibroblasts, indicating that stromal states are also maintained by coordinated regulatory networks (Li M. et al., 2026). As spatial transcriptomics and multi-omics integration advance, GRN inference is expected to incorporate cell neighborhoods and intercellular communication (Li S. et al., 2026).

Incorporating the microenvironment will require methods that separate cell-intrinsic regulation from ligand-receptor-driven effects and that account for spatial proximity when assigning regulatory influence. These extensions are likely to change how GRNs are defined in complex tissues.

The cancer studies discussed above differ substantially in the strength of evidence they provide. In mouse skin squamous cell carcinoma, paired scRNA-seq and scATAC-seq identified EMT-state regulators, and CRISPR/Cas9 experiments supported roles for Klf5, Pitx1, Nfatc1, and Creb3l1 (Perez-Gonzalez et al., 2026). In patient-derived glioma stem-like cells, network topology informed resistance trajectories and siRNA or combination-treatment simulations, but these findings remain preclinical (Park et al., 2024). In melanoma, a TCF4-dependent GRN was linked to immunotherapy resistance and supported by functional perturbation in melanoma models (Pozniak et al., 2024), whereas network-based resistance-state diagrams remain primarily computational (Pillai et al., 2022). In hepatocellular carcinoma, terminal-state signatures were associated with survival but have not been prospectively validated (Deng et al., 2026). Other studies in leukemia, lung adenocarcinoma, renal cell carcinoma, prostate cancer, gastric cancer, and the tumor microenvironment provide retrospective or preclinical evidence rather than established clinical utility. Differential-expression analysis alone would not resolve regulatory wiring, directionality, state transitions, or perturbation sensitivity; these are the specific contributions of GRN inference.

## 5 From prediction to validation: interpretation and causal verification

A practical workflow links network inference, *in silico* perturbation, and experimental validation. Whether these predictions can be verified determines whether GRN models can move toward translation. A mature workflow is network inference, virtual perturbation, and experimental validation: CellOracle, Pando, GenKI, and scTenifoldKnk generate *in silico* perturbation candidates (Osorio et al., 2022; Fleck et al., 2023; Kamimoto et al., 2023; Yang et al., 2023), while Spear-ATAC and CAT-ATAC provide single-cell perturbation readouts (Pierce et al., 2021; Shevade et al., 2025). This loop has already uncovered regulatory dependencies in glioblastoma, melanoma, and prostate cancer organoids.

In these settings, the loop has identified regulators whose loss changes cell state, and it provides a template for validating predicted dependencies before more expensive *in vivo* experiments. The same framework can be extended to patient-derived organoids and primary samples, where perturbation screens are more difficult but clinically more relevant.

Validation results need careful interpretation. A perturbation experiment tests gene function in a specific context, while a GRN edge predicts a relationship in that context. Agreement strengthens the edge; disagreement may reflect the network, the perturbation design, or the cell state itself, and should be reported rather than hidden.

Foundation models add new capabilities and new interpretability questions. Single-cell and epigenomic foundation models are changing GRN inference. GET learns regulatory grammar across large collections of human fetal and adult cell transcriptomes (Fu et al., 2025). EpiAgent, pretrained on scATAC-seq, encodes cells as concise “cell sentences” and supports *in silico* knockout of cis-regulatory elements (Chen et al., 2025). RegFormer embeds GRN priors into a Mamba architecture, is pretrained on 25 million human single cells, and improves perturbation response and drug response prediction (Hu et al., 2026). ChromBERT learns interpretable context-specific regulatory network representations from ChIP-seq profiles of roughly 1,000 transcription regulators (Yu et al., 2026). These models may alleviate sparsity and improve generalization across cell states (Yiu et al., 2025; Li S. et al., 2026).

The interpretability of these models requires careful evaluation. A systematic evaluation found that attention-derived edge scores from scGPT and Geneformer add little beyond gene-level statistics for perturbation-target prediction (Kendiukhov, 2026c). Causal circuit tracing showed that relations between internal model features are not the same as biological causation (Kendiukhov, 2026a). An analysis of GRN evaluation metrics found severe label missingness and study bias in reference networks, leaving foundation-model scores far below the observable ceiling (Kendiukhov, 2026b). Attention weights, gradient attributions, and model-internal circuits should therefore be treated as hypothesis generators rather than causal evidence.

Foundation models should be integrated into the methodological taxonomy rather than treated as a separate late-stage topic. Their potential advantages include transfer learning, cross-cell-type generalization, regulatory representation learning, and improved handling of sparse observations. Their limitations include pretraining bias, domain shift, data leakage, high computational cost, weak mechanistic interpretability, and uncertainty about whether learned attention or embedding structure corresponds to biological regulation. Foundation-model outputs should therefore be compared with conventional graph, generative, and co-expression baselines and validated with perturbation assays.

These limitations do not make foundation models unsuitable for GRN inference, but their outputs need to be validated with perturbation data and compared with simple baseline features. Until such validation becomes standard, model-derived regulatory edges should be reported with the same caution as any other computational prediction.

## 6 Controversies, challenges, limitations, and future directions

The central unresolved issue is causation: most GRN methods infer statistical associations rather than regulatory relationships, and even when chromatin accessibility, motifs, and footprints are included, directionality, feedback loops, and enhancer-promoter causality still require perturbation experiments (Badia et al., 2023; Loers and Vermeirssen, 2024). Virtual perturbation offers low-cost candidates, but its predictions must be checked against real CRISPR phenotypes across more tumor models (Kamimoto et al., 2023; Yang et al., 2023). This distinction matters clinically because a highly connected regulator is not necessarily a good drug target if its loss has no phenotypic effect; a regulator that drives resistance and one that merely marks it demand different actions, so computational models should separate these possibilities while treating validation design as part of the inference pipeline.

Evaluation standards are also unsettled. Methods are accumulating faster than benchmarks, and differences in reference networks, preprocessing pipelines, and metrics make cross-study comparison difficult (Badia et al., 2023). Partial labels, study bias, and untested interpretable outputs such as attention edge scores can inflate apparent performance unless evaluation specifies label coverage, observable ceilings, and incremental-value tests (Kendiukhov, 2026c; Kendiukhov, 2026b). Clinical translation is further constrained by scarce paired clinical samples, batch effects, tumor purity, and the still-technical status of deep-learning interpretation of chromatin and methylation data (Yiu et al., 2025; Loers and Vermeirssen, 2024; Rotkevich et al., 2025; Aref-Eshghi et al., 2025); progress therefore depends on datasets that link molecular profiles to outcomes, methods robust to technical noise, and validation studies in appropriate model systems.

Progress will require coordinated efforts in four areas: multimodal expansion that adds DNA methylation, histone modifications, spatial position, and temporal dynamics to transcriptome and accessibility data (Badia et al., 2023; Loers and Vermeirssen, 2024); causal AI that combines foundation models, virtual perturbation, and high-throughput CRISPR screens to distinguish causation from correlation (Kamimoto et al., 2023; Shevade et al., 2025); unified benchmarks spanning tumor types, platforms, and perturbations with explicit treatment of label missingness and metric ceilings (Kendiukhov, 2026b); and clinical translation that maps cell-state-specific GRNs to drug response, immunotherapy efficacy, and combination targeting (Li S. et al., 2026; Yiu et al., 2025). These directions are interdependent: multimodal data improve causal inference, causal inference improves benchmark design, and reliable benchmarks are prerequisites before clinical decisions can be based on network predictions.

The challenges discussed here fall into two groups. Some are directly addressable through better methods and reporting: three-dimensional enhancer-promoter looping, uncertainty quantification and calibration, reproducibility, code availability, hyperparameter sensitivity, negative controls, and benchmark design. Others reflect broader biological and clinical complexity: tumor purity, immune and stromal composition, spatial organization, clonal architecture, treatment history, cell-cycle state, and batch or platform effects. The first group can be improved through methodological and reporting standards; the second requires careful study design and biological validation.

This article is a narrative mini-review rather than a systematic review, and several limitations should be acknowledged. The literature search was not exhaustive and may be affected by selection and publication bias, English-language bias, and the rapid obsolescence of AI methods. We did not perform a quantitative meta-analysis, and several cited methods lack independent replication. Preprints were included when directly relevant and are labelled as such. The comparison should therefore be read as a structured expert synthesis rather than a formal meta-analysis.

Several priorities follow from these gaps. First, benchmarks should span tumor types, platforms, and perturbations while explicitly reporting label coverage and metric ceilings. Second, multimodal data should be integrated with causal perturbation readouts. Third, uncertainty quantification and interpretability of foundation models need improvement. Finally, candidate regulators should be validated in patient-derived organoids, primary samples, and clinically annotated cohorts.

## 7 Conclusion

Paired single-cell transcriptome and chromatin accessibility data provide same-cell correspondence between regulatory elements and transcriptional output, and AI and computational methods provide the core tools for learning cell-state-specific networks from sparse, high-dimensional data. SCENIC+, scMEGA, STREAM, scMTNI, LINGER, scMultiomeGRN, DeepMAPS, and Dictys show the methodological value of multi-omics integration, while CellOracle, Pando, Spear-ATAC, and CAT-ATAC connect network predictions to experimental validation. Applications in EMT, resistance, lineage plasticity, and subtype analysis show that GRNs can move from descriptive atlases toward testable models. The next step is not more architectures but shared benchmarks, stronger causal validation, and interpretable, verifiable models.
